# Remote Sensing and Assessment of Compound Groundwater Flooding Using an End-to-End Wireless Environmental Sensor Network and Data Model at a Coastal Cultural Heritage Site in Portsmouth, NH

**DOI:** 10.3390/s24206591

**Published:** 2024-10-13

**Authors:** Michael R. Routhier, Benjamin R. Curran, Cynthia H. Carlson, Taylor A. Goddard

**Affiliations:** 1 Institute for the Study of Earth, Oceans and Space, University of New Hampshire, Durham, NH 03824, USA; 2 Forensis Building Conservators, Baltimore, MD 21208, USA; forensisbc@gmail.com; 3 Department of Civil Engineering, Merrimack College, North Andover, MA 01845, USA; carlsonc@merrimack.edu

**Keywords:** cultural heritage, historic preservation, coastal flooding, climate change, groundwater, water level monitoring, streaming data, wireless sensor networks, data dashboard

## Abstract

The effects of climate change in the forms of rising sea levels and increased frequency of storms and storm surges are being noticed across many coastal communities around the United States. These increases are impacting the timing and frequency of tidal and rainfall influenced compound groundwater flooding events. These types of events can be exemplified by the recent and ongoing occurrence of groundwater flooding within building basements at the historic Strawbery Banke Museum (SBM) living history campus in Portsmouth, New Hampshire. Fresh water and saline groundwater intrusion within basements of historic structures can be destructive to foundations, mortar, joists, fasteners, and the overlaying wood structure. Although this is the case, there appears to be a dearth of research that examines the use of wireless streaming sensor networks to monitor and assess groundwater inundation within historic buildings in near-real time. Within the current study, we designed and deployed a three-sensor latitudinal network at the SBM. This network includes the deployment and remote monitoring of water level sensors in the basements of two historic structures 120 and 240 m from the river, as well as one sensor within the river itself. Groundwater salinity levels were also monitored within the basements of the two historic buildings. Assessments and model results from the recorded sensor data provided evidence of both terrestrial rainfall and tidal influences on the flooding at SBM. Understanding the sources of compound flooding within historic buildings can allow site managers to mitigate better and adapt to the effects of current and future flooding events. Data and results of this work are available via the project’s interactive webpage and through a public touchscreen kiosk interface developed for and deployed within the SBM Rowland Gallery’s “Water Has a Memory” exhibit.

## 1. Introduction

### 1.1. Background

This research evaluated the use of an end-to-end water level sensor network (sensor data collection to public consumption of data) to “remotely sense” the groundwater intrusion rates within historic building basements at the Strawbery Banke Museum (SBM) coastal cultural heritage site in Portsmouth, New Hampshire. This work was completed in order to measure, assess, and model the timing, quantities, and influences of contributing drivers of groundwater flooding and to promote more informed flood adaptation, mitigation, and public engagement activities.

Cultural heritage sites are important assets to humanity because they can help define a community’s identity, promote diversity, educate the public, contribute to a region’s economy, provide creative inspiration to the arts, and encourage tourism [1,2]. Low-elevation coastal zones (LECZs), less than 10 m above sea level, have been the focus of human settlement for thousands of years and are home to some of the world’s most important cultural heritage sites [3,4]. However, ongoing increases in sea levels and coastal flooding events driven by climate change are putting these sites at risk of accelerated deterioration and destruction [5,6,7]. Along US coastlines alone, current 50-year extreme water levels are expected to be exceeded annually by 2050 [8], and current 100-year extreme water levels are expected to increase in frequency by 40-fold over the same time period [9].

Flooding events that are caused by multiple environmental drivers, such as rainfall, king tides, and storm surges happening concurrently or in sequence, are known as compound flooding events [10,11]. These events, due to their boosted ability to increase the height of local water tables due to the combined effects of tidal forcing and rainwater saturation of soils [12,13,14,15], put local buildings at risk due to the direct and indirect effects of basement inundations [16,17]. Direct effects of basement inundation include damage caused to a property through direct contact with waters during a flooding event [17], such as damage to basement furnaces or water heaters. Indirect effects of basement inundations can include the warping of joists, panels, and flooring; rusting of fasteners; deterioration of foundations and mortar; and mold growth within walls due to exposure to dampness and increased humidity throughout affected structures [16,17,18,19,20]. Other indirect effects of increased moisture due to groundwater inundation can include the potential destabilization of buildings due to the growth of brown and white rot fungus in wooden structural members that contact foundations, such as sills, plates, and posts [21,22,23]. These moisture conditions can also promote infestations of invasive termites that have an increasing potential for the destruction of historic buildings as they migrate northwards with long-term regional temperature increases [24,25,26,27]. Furthermore, the accumulation of salts in, on, and between materials, known as efflorescence, can result in the delamination of finishes [28,29,30], surface marring, corrosion of materials [31], and the internal structural degradation of bricks and mortars due to expansive forcing [32,33].

Recent studies involving locations with distinct hydrological and geological characteristics have highlighted the importance of incorporating compound drivers into the design of flood assessments [7,34]. For instance, Rahimi et al. (2020) [34] modeled local compound flooding events in the coastal San Leandro watershed of Oakland Flatlands, California, to assess the capacity of existing drainage infrastructure within constituent communities. Results showed that the combined effects of sea-level-rise (SLR), groundwater inundation, and precipitation could potentially flood up to 283 hectares (700 acres) of the area’s built infrastructure during compound flooding events, a substantial increase over model scenarios that did not take into account compound flooding. Further, Wahl et al. (2017) [7] evaluated literature about the uncertainties of extreme sea levels (ESLs) caused by the combination of normal wave action, extreme tides, and storm surges beyond just projected sea levels to understand coastal flood risks better globally. Results showed that both ESL estimates and SLR projections were needed to estimate the future risk of coastal flooding better.

With regard to coastal cultural heritage buildings, assessments of compound flooding may have implications with respect to those buildings’ future adaptation and mitigation strategies. For instance, basements that are more inundated with freshwater from rainwater saturation of surrounding soils might require the use of sump pumps and a dehumidifier to help mitigate the effects of the flooding and its associated humidity [35]. However, basements more inundated with salt water may also require the replacing of foundations with materials like corrosion-resistant concrete that are less susceptible to salt water corrosion [36] or types of granite that are less susceptible to wicking than other materials such as bricks [37].

In order to assess environmental phenomena, such as groundwater inundation levels remotely at cultural heritage sites or any locations, it is first necessary to collect data associated with these phenomena at those locations. One way to do this is with the use of remote wireless environmental sensors and their associated networks [38]. Wireless environmental sensor networks consist of (1) a series of sensor nodes that measure and collect data autonomously, (2) a communications system that transmits the collected data, and (3) a cloud and network server that stores the data [39]. Some networks also distribute and visualize their collected data via public-facing web page interfaces, essentially completing an end-to-end network from data collection to public consumption of measured environmental data. Some such public interfaces within end-to-end environmental sensor networks include the Southeast Coastal Ocean Observing Regional Association (SECOORA), Southeast Water Level Network (SWLN) [40], and the USGS National Water Network [41]. The SWLN utilizes over 100 water level sensor stations and a public web interface for live flood hazard monitoring and flood alerts to help promote community resilience and preparedness [40]. The USGS National Water Network web interface provides real-time distributions and visualizations of sensor water level data, weather data, and flood forecasts from more than 13,500 USGS observation stations across the country. The USGS National Water Network interface supports local and national decision making, emergency management, and public safety operations during important hydrological events such as droughts and floods [41].

An assessment of previous research literature reveals several studies that have used remote wireless environmental sensors to collect and monitor surface water level flooding data [42,43]. For instance, Loftis et al. (2018) [42] deployed a series of streaming water level (pressure) monitoring sensors along the coast in Hampton Roads, Virginia, to help establish a regional resilience monitoring network for the area. The data was used to help drive a street-level flood model that was then validated with crowd-sourced data and a USGS water level station in 2017 during Hurricanes Jose and Maria. Further, Mendoza-Cano et al. (2021) [43] developed a wireless sensor network for the collection of inland data for the city of Colima-Villa de Álvarez, Mexico. The Colima-Villa de Álvarez network collected hydrometeorological, fluvial water-level, and soil moisture data that were then used to create a hydrological model and flood inundation maps to help identify at-risk infrastructure.

Other studies have used remote wireless environmental sensors to more specifically collect, monitor, and assess groundwater-level data [44,45]. For instance, Xue et al. (2010) [44] demonstrated how to build an environmental sensor network for real-time monitoring and distribution of current and historical groundwater data for the State of Nebraska. The data from the Nebraska network has since been utilized to create a state-wide groundwater map and was to be added later into the National Drought Information System (NIDIS) to support future regional and national decision making. Further, Knott et al. (2019) [45] utilized water level data from nearly 3000 groundwater sites, in conjunction with other hydrologic and landscape characteristic data, to model changes in sea-level-rise-induced groundwater levels across seacoast New Hampshire. With the use of the seacoast New Hampshire model, Knott et al. (2019) [45] projected how far inland sea-level rise was affecting the rise of groundwater and discussed what the implications of this rise could be over time.

Environmental sensors have also been used in research studies to monitor environmental phenomena at cultural heritage sites [46,47]. For instance, Grammalidis et al. (2011) [46] created a wireless environmental sensor network for use in the Mediterranean region to remotely monitor archaeological and cultural areas of interest at risk from wildfires. The network utilized infrared cameras and temperature sensors as part of an early warning system for the sites. While Klein et al. (2017) [47] utilized a network of 200 temperature, humidity, and air pressure sensors at the medieval branch of the New York Metropolitan Museum of Art to help maintain the ideal preservation conditions for the art on display. This network monitored and assessed the overall climate of the museum to better understand how changes in micro-climates may occur due to drivers, such as the arrival and distribution of visitors throughout the facility.

However, despite the plethora of examples of environmental sensor networks being used to monitor surface and groundwater flood levels along our coasts and in-land and environmental hazards at cultural heritage sites, there appears to be a dearth of research studies that use environmental sensors to monitor and assess groundwater inundation flood risks within the basements of coastal cultural heritage buildings. The only published journal study that was found as an exception to this dearth collected and assessed groundwater inundation data within the basements of the coastally situated Strawbery Banke Museum (SBM) in Portsmouth, New Hampshire, in 2012 [48]. This 2012 SMB study, completed by some of the same authors as the current study, showed that the compound drivers of precipitation and tides influenced groundwater flood levels within some of the buildings across the museum’s campus. As the 2012 SBM study utilized static water level sensors whose data were downloaded in-person only once per month, the network was useful for documenting previous flood events but lacked the data download cadence to also act as a live-streaming system to monitor flood events in near-real time. Also, the 2012 study, which only measured water levels with regard to its compound basement flood drivers, lacked the ability to measure the concentrations of dissolved salts in the groundwater. Furthermore, as the 2012 study did not implement a public-facing interface, it lacked the ability to distribute and visualize live streaming data for public consumption and local on-the-fly decision making. The limitations of the 2012 SBM static sensor study helped to form the impetus and foundation for the implementation of the present SBM end-to-end streaming wireless water level sensor network and its accompanying study.

### 1.2. Objectives

The objectives of this study were to:

(1) Build an end-to-end groundwater sensing network at the Strawbery Banke Museum coastal cultural heritage site.
(2) Assess variations in the timing and amount of groundwater intrusion and salinity levels within historic buildings at the Strawbery Banke Museum.
(3) Develop simple statistical models to understand better and parse the levels of influence that tidal forcing and rain event drivers have on groundwater basement flooding at the Strawbery Banke Museum.
(4) Promote public engagement and community decision making about coastal flooding and adaptation and mitigation strategies.

### 1.3. Hypotheses

Because of the importance of coastal cultural heritage sites, the ongoing and increasing threats of climate change, and the proven abilities of previous environmental sensor surveys to monitor flooding in other at-risk locations around the world, our research looks to find new ways to utilize a sensor network to detect and assess basement groundwater inundations at the Strawbery Banke Museum coastal cultural heritage site. To this end, we hypothesize that:

**Hypothesis 1 (H1).**  *A wireless groundwater environmental sensor network can be built within the complex hydrological environment of the Strawbery Banke Museum coastal cultural heritage site.*

**Hypothesis 2 (H2).**  *Variations in the timing and amount of groundwater intrusion levels and water salinity levels can be measured with a wireless latitudinal sensor network within the historic building basements at the Strawbery Banke Museum.*

**Hypothesis 3 (H3).**  *Simple statistical models can be utilized to help parse the levels of influence that tidal and rain event drivers have on compound groundwater basement flooding levels at the Strawbery Banke Museum.*

**Hypothesis 4 (H4).**  *An interactive public-facing web page interface can be built to support public engagement and local decision making with regard to groundwater inundation at the Strawbery Banke Museum.*

## 2. Materials and Methods

### 2.1. Study Area

**Figure 1:**
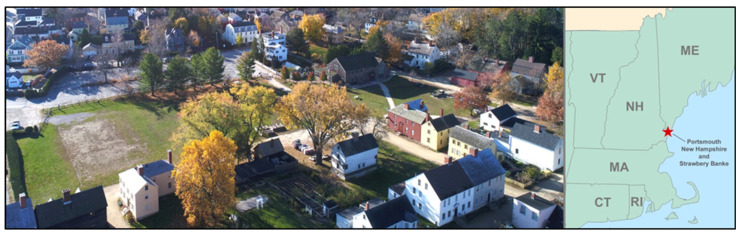
Aerial Image looking south over the central green and a portion of the historic houses on the Strawbery Banke Museum campus in Portsmouth, New Hampshire. (Photo by: Taylor Goddard © 2023).

This work was conducted at the Strawbery Banke Museum (SBM) in Portsmouth, New Hampshire (USA) at approximately −77.75° W Longitude by 43.07° N Latitude (Figure 1). This museum is a 4-hectare (10-acre) living history museum that contains 40 buildings from the colonial and pre-colonial eras. The museum campus is situated within 366 m (1200 feet) of the nearby Piscataqua River tidal estuary that drains into the Gulf of Maine and the greater Atlantic Ocean. At the center of the SBM campus is a large 1-hectare (2.5-acre) central green area that extends east through Portsmouth’s Prescott Park, lying adjacent to the Piscataqua River. This green space in colonial and post-colonial times was open to the river as a tidal inlet and dock area known as Puddle Dock. Starting in the early 1900s, Puddle Dock was filled in with loose fill and debris from the surrounding community to form the central green area that exists today. However, engineering surveys [49], previous sensor deployments on-site [48], and empirical observations of puddled water in low-lying areas on its surface during very high tide events suggest that tidally induced groundwater flooding might still be occurring on the site.

### 2.2. The Network and Assessment

The methods employed within this study included (1) the design, deployment, and use of an end-to-end environmental sensor network and (2) the assessment and modeling of the network’s collected data.

#### 2.2.1. The Network Design, Deployment, and Use

**Figure 2:**
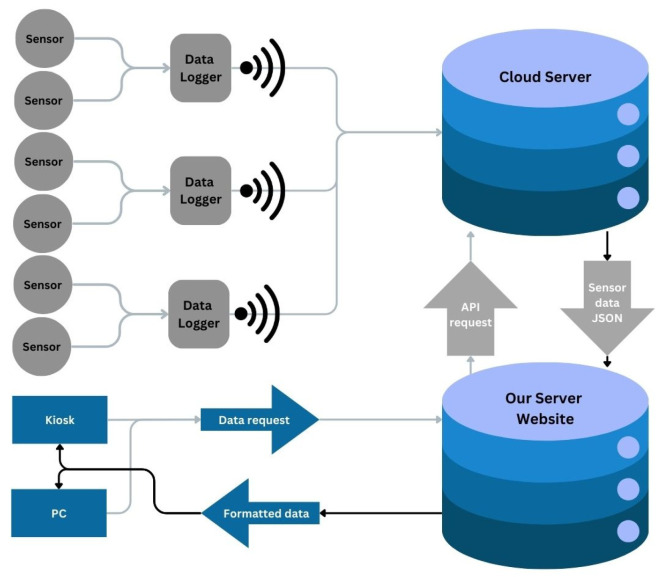
Diagram of the Strawbery Banke Museum Environmental Sensor Network consisting of sensors, data loggers, a cloud server, a data and web server, and public-facing interfaces.

The network itself consists of hardware, software, and transmission protocols for a series of sensors, data loggers, wireless transmitters, a cloud server, a local data and web server, and two public-facing interfaces (Figure 2).

**Figure 3:**
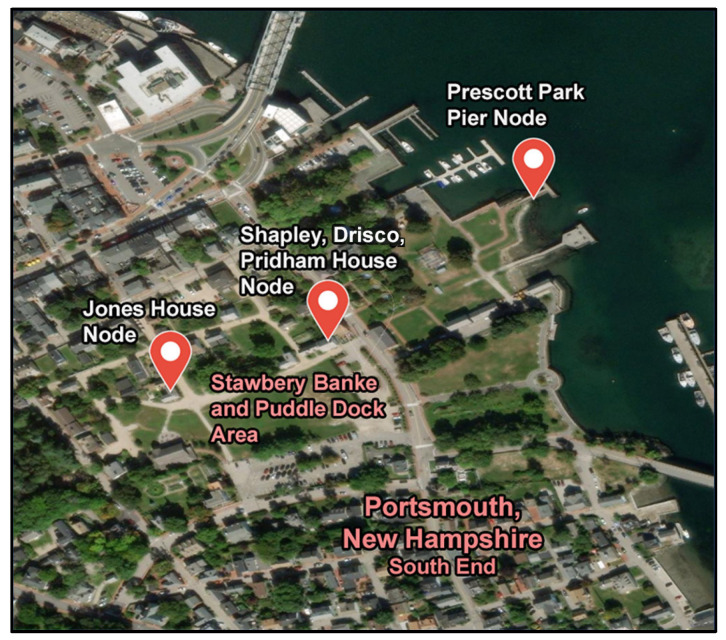
Map of Strawbery Banke Museum Environmental Sensor Node Network within the South End of Portsmouth, New Hampshire.

To date, three environmental sensor nodes consisting of one or two sensors each have been deployed on-site at SBM (Figure 3). These include one in a sump pump pit in the Jones house basement, located approximately 244 m (800 feet) from the tidally influenced Piscataqua River, another in a sump pump pit in the Shapley, Drisco, Pridham (SDP) house basement, approximately 122 m (400 feet) from the river, and a third off of the Prescott Park Pier, sitting directly in the tidal river itself. These locations were chosen because basement flooding had been regularly observed within these buildings during large tidal or prolonged rain events prior to the installation of the network because the locations are approximately parallel to the location of the old Puddle Dock Inlet, suspected to be the conduit of tidal forces on local groundwater and because the locations mimic the sensor deployment locations of a previous one-year 2012 static sensor network deployment. All horizontal and vertical sensor positions in the new network were surveyed with the use of a Trimble (Westminster, CO, USA) TSC7 data collector [50] and an R12i receiver [51] for measurements made above grade and a conventional measuring tape for measurements made below grade. The survey utilized a NAD83 horizontal datum and a NAVD88 vertical datum. The Trimble R12i receiver being used in Portsmouth, NH, 16 km away from its base station in Durham, NH, is estimated to have a 16 mm horizontal accuracy and a 23 mm vertical accuracy [51].

**Figure 4:**
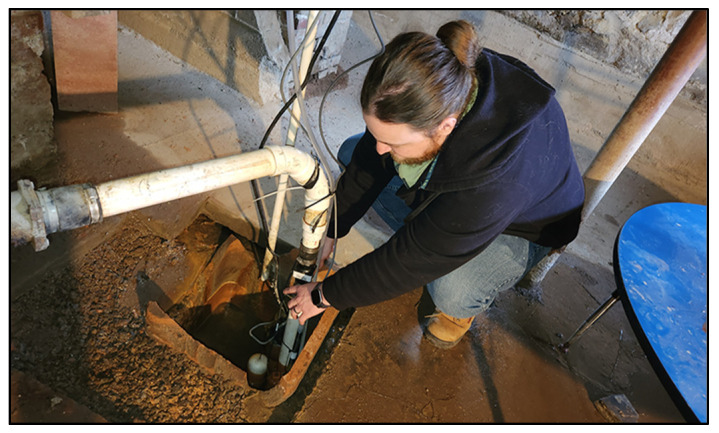
Installation of an MX2001-04-TI-S OnSet© water level sensor and a pHionics© STs series conductivity/salinity sensor located along the side of a drain pipe within a sump pump pit in the Jones house basement.

The Jones house node consists of an MX2001-04-TI-S OnSet© (Bourne, MA, USA) water level sensor [52] and a pHionics© (Antioch, CA, USA) STs series conductivity/salinity sensor [53] located within a sump pump pit in the building’s basement (Figure 4) and connected to an AC-powered OnSet© RX3004 data logger [54] above grade via a 9 m transmission cable. The Shapley, Drisco, Pridham (SDP) house node consists of a similar setup of an MX2001-04-TI-S OnSet© water level sensor and a pHionics© STs series conductivity/salinity sensor located within a sump pump pit in the basement and connected to an AC-powered OnSet© RX3004 data logger [54] above grade via transmission cables. The Prescott Park pier node consists of a single OnSet© water level sensor [52] located below grade at the base of one of the support pilings of the pier and is connected to a 5w solar panel powered OnSet© Microstation data logger [55], located above grade via a 9 m transmission cable. Sensors and cables in the Jones and SDP house nodes were secured using zip ties to vertical sump pump drainage pipes extending upward from each basement’s sump pits. The Prescott Park Pier sensor and its accompanying cable were threaded through a 2.5 cm (1-inch) wide PVC conduit and secured to one of the pier’s support pilings using a series of zip ties at 1 to 2 m increments upwards from the sensor.

The OnSet© water level sensors [52] measure water temperature, water pressure, and water level. The pHionics salinity sensors [53] measure water conductivity, water temperature, and water salinity. All of the OnSet© water level sensors [52] used on the project each hold 3-point National Institute of Standards and Technology (NIST) traceable calibration certificates from when purchased and have a reported accuracy of ±0.3 to 0.6 cm of water level accuracy. The pHionics© salinity sensors [53] were calibrated on-site prior to operation using a standard seawater 35 ppt salinity solution as recommended by the manufacture and have an estimated accuracy of 1 to 1.5% of the measured salinity.

The Jones and SDP house data loggers were placed above grade on the first floor of each house and hidden from direct public view in a closet and behind a historical display, respectively. The Prescott Park Pier data logger was placed beyond arm’s reach in a locked stainless steel box, secured to a plywood panel, facing outward from the wood railings of the pier. The data logger’s solar panel was mounted flush next to the stainless steel box on the plywood panel, also beyond arm’s reach. These placements were chosen to help dissuade vandalism or tampering with the box, data logger, and solar panel, as the pier is frequented by many guests annually.

**Figure 5:**
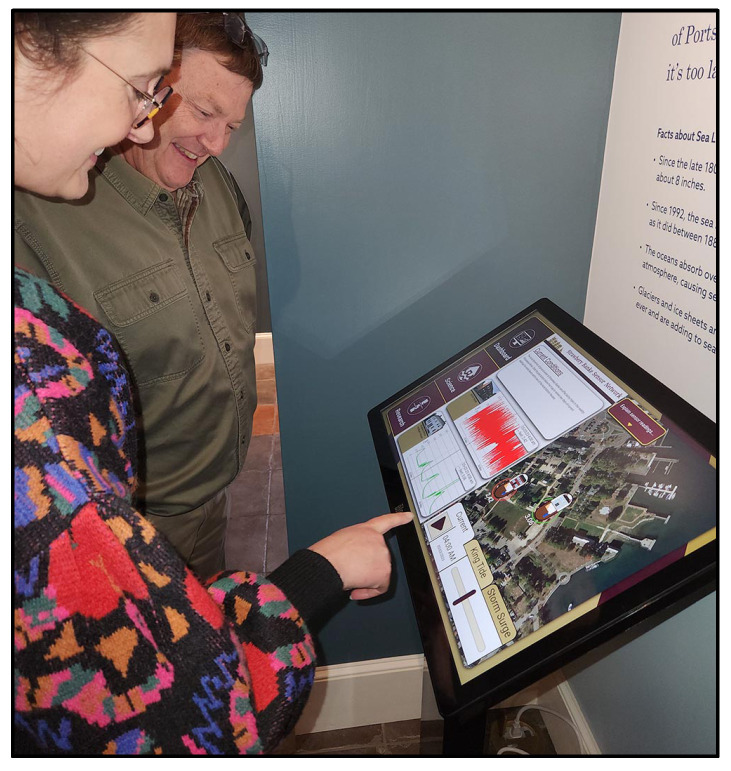
Image of Rodney Rowland, Director of Environmental Sustainability, and Dr. Alix Martin, Archeologist of the Strawbery Banke Museum, utilizing its Sensor Network Touch Screen Kiosk found in the Rowland Gallery, “Water Has a Memory” exhibit.

Sensor measurements at each data logger are collected and stored with a typically 5 min cadence but varied between 1 and 5 min during the early installation of the sensors. The wireless transmission of stored data is regularly pushed to a cloud server database every 10 min from the data logger, using an onboard cellular node and an AT&T 4G network [56]. Data stored on the cloud drive is collected regularly using a remote API request and processed on a local data and web server equipped with Intel(R) Xeon(R) CPU E5-2643 0 @ 3.30 GHz 128 GB RAM and a Matrox Electronics Systems Ltd. (Maxtrox Video, Dorval, QC, Canada) G200eR2 GPU running an Ubuntu operating system (Canonical, London, England) [57] and a PostgreSQL database (Global Development Group, Brisbane, Australia) [58]. Data as parsed from the database are then used to populate a project-created web-mapping interface that utilizes a Django framework [59] and an Open Layers web-mapping library [60]. This interface was created for the remote presentation of the data over the Internet to engage the public about the risks of groundwater flooding and to help in community engagement and decision making. The interface features a dashboard panel for the browsing, mapping, and graphing of current two-day water level conditions across the SBM sensor network, as well as examples of historical storm surge and king tide scenarios that can be displayed as if in accelerated historical time with a time-slider tool. To further facilitate public engagement with the data, a special version of the web interface was also created to function on a touchscreen kiosk located in the Rowland Gallery at the SBM. This special interface contains larger icons, graphics, buttons, and controls to help facilitate the use of the touchscreen. The kiosk itself is created with an AC-powered ELO (Suzhou, China) 0.69 m (27-inch) touchscreen [61] attached to a 1.5 m (60-inch) tall free-standing base. The touchscreen is driven by an ELO Backpack 4 computer system [62] attached to the back of the display and runs an Android 10 operating system (Google, Mountain View, CA, USA) [63] (Figure 5). Both the ELO touchscreen and the ELO Backpack 4 are powered via a standard AC power outlet and are connected to the Internet via the Rowland Gallery’s Wi-Fi network.

In addition to the data we collected with our network, we also utilized NOAA weather station hourly precipitation data. The Pease Air Force Base, Portsmouth, NH, station (Station Name: PORTSMOUTH PEASE AFB, NH US; Network ID: WBAN:04743) [64] was selected as the closest, most complete data set for the time period. The rain gauge is approximately 5.1 km (3.2 miles) from the Strawbery Banke campus.

#### 2.2.2. Data Assessment

*Data Preparation*:

The raw data were first checked and cleaned to ensure that they were consistent within sensors and across sensors. Examples of issues observed with the sensors include change in datum between initial installation and final surveyed location, temporary loss of signal due to prolonged power outages beyond the charge of the data logger batteries, and change in cadence of data collection (i.e., one minute vs. five minutes). As our data set was not prohibitively large, manual cleaning, recognized as the most accurate cleaning method [65], was possible. Erroneous data, due to factors such as network outages, were discarded, and irregular time steps were averaged to result in standard 5 min and daily time steps.

*Timing and Quantities*:

Next, in order to better understand the behavior of the project’s collected water level and groundwater salinity sensor data, we assessed it for variations in time and quantities over the collection periods at both the SDP and Jones houses. Time lags and quantities were estimated where possible between peak tides in the nearby Piscataqua River and peak water level inundations at each of the houses.

*Modeling Component Flooding*:

Next, a series of simple linear models for basement flooding were created under varied compound rainfall and tide scenarios to assess the influences of individual and compound drivers. These included water level data versus:

- All high tides;
- High tides > 1.2 m (4 feet);
- Precipitation alone;
- Precipitation and all high tides;
- Precipitation and high tides > 1.2 m (4 feet).

For each model, the following statistics were calculated at both the SDP and the Jones houses.

1. R^2^ to estimate the influence of each flood driver.
2. *p*-value to measure the likelihood that any observed correlation between water level, seepage, tidal levels, and precipitation are occurring by random chance.
3. Slope to enable prediction of future water levels at each house.
4. BP value and associated *p*-value to measure the skew/heteroscedasticity of the model’s error term residuals.

We currently opted to use this simple linear model within our study instead of machine learning or more complex modeling techniques for two primary reasons. First, the simple model effectively captures the relationships present in the data. Second, for the model to be practical for managers of historic buildings, it must remain accessible without requiring specialized software or advanced expertise.

## 3. Results

### 3.1. Time and Quantity Results

#### 3.1.1. Piscataqua River Time and Quantity Results

**Figure 6:**
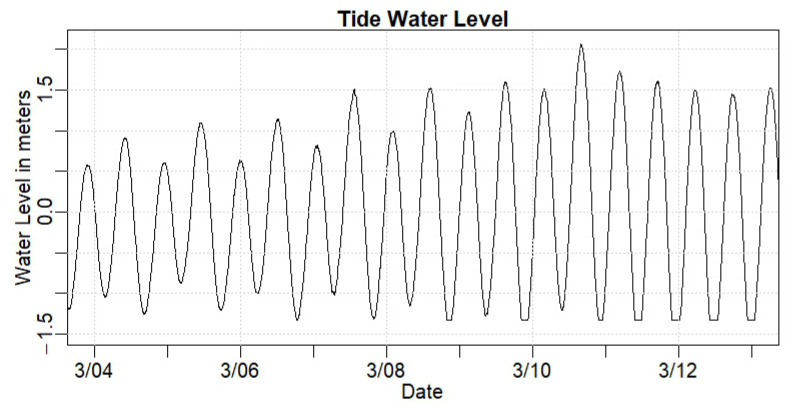
Graph portraying typical tidal variation in water level at the Piscataqua River sensor node. High tides vary from approximately 0.5 m above mean water to over 2 m above mean water.

Figure 6 shows the water level at the Piscataqua River meter from 3 March 2024 to 13 March 2024. The location experiences approximately two tides daily, with high tides generally over 0.5 m above mean sea level and some higher high tides of over 2 m. Note that during March of 2024, the start of the month shows relatively low high tides, and the highest monthly higher high tide occurred in the late evening of March 10. This period, therefore, is helpful to illustrate the influence of tidal forcing at the historic properties. While statistical analysis is based on our whole period of record (12 December 2023–31 July 2024), this period at the beginning of March 2024 was used to visualize the influence and results.

#### 3.1.2. Shapley Drisco Pridham (SDP) House Time and Quantity Results

**Figure 7:**
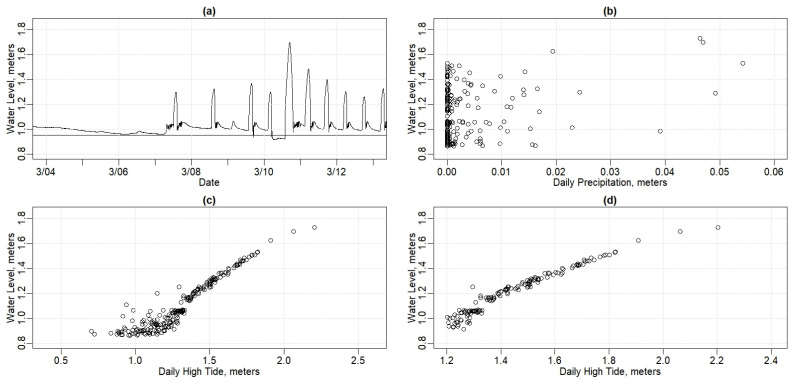
Graphs portraying Shapley Drisco Pridham (SDP) house. (**a**) Basement water levels from 3 March to 13 March 2024. (**b**) Daily average basement water levels vs. daily sum of precipitation. (**c**) Daily average basement water levels vs. daily high tide. (**d**) Daily average basement water levels vs. daily high tides > 1.2 m.

The SDP house, approximately 122 m (400 feet) from the tidal Piscataqua River, showed a strong relationship between flooding and higher tide levels. Figure 7a shows the water level measured in the SDP house basement for the same time period as shown in Figure 6. Note that there is very little indication of water entering the SDP basement from 3 March to 7 March, after which there is approximately a 0.3 m (1-foot) increase in basement water level once daily. Starting on 9 March, there is a small tidal response for the morning high tide, which resolves to two responses daily, one for each high tide, from 10 March to 12 March. The flooding response is apparently occurring for tides greater than 1.2 m (4 feet), while very little response is seen for lower high tides.

Figure 7b compares the basement water level in the SDP house to the recent daily total precipitation from 12 December 2024 to 31 July 2024. There is little to no apparent relationship. This, in contrast to the apparent clear impact of the tidal forcing, leads us to conclude that the SDP house flooding is largely driven by tides.

Figure 7c shows the relationship between the SDP basement water level and tides more clearly. This figure shows, for each day from 14 December 2023 through 31 July 2024, the daily highest water level plotted against the daily highest tide. At a higher high tide level, above approximately 1.2 m (4 feet), the water level in the SDP house is nearly linear (Figure 7d, R^2^ = 0.94), while at lower tide heights, the basement does not exhibit as much flooding response. This may be an artifact of the pump being able to maintain a lower water level for lower tides or that the lower tides do not force as much water into the groundwater as do the higher tides. Note that the highest daily higher high tide (2.4 m, 8.71 feet) paired with the highest basement water level (1.92 m, 6.38 feet) occurred on 13 January 2024, the storm with record flooding at SBM. The lag time between the higher high tide in the Piscataqua River and the peak of water level in the SDP house basement is approximately one hour.

**Figure 8:**
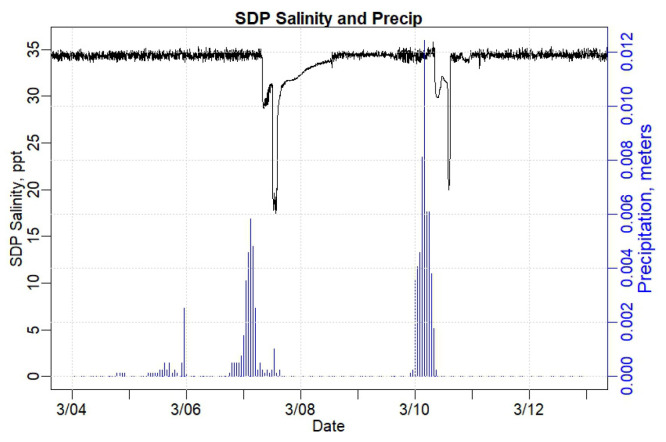
Graph portraying Shapley Drisco Pridham (SDP) house salinity levels (black, left axis) in basement water and precipitation (blue, right axis) measured at the Pease Air Force Base, Portsmouth, NH.

Figure 8 shows the salinity measured in the SDP house basement (black, left y-axis) over time and the precipitation (blue, right y-axis) for the same time period, as shown in Figure 6, 3 March to 13 March 2024. The salinity does not vary widely with tide, remaining largely between 34 ppt and 35 ppt—which is typical for pure ocean water. Note that when the salinity in the basement of the SDP house falls, it generally coincides with a precipitation event; however, there are precipitation events that do not seem to cause a change in the salinity in the SDP house. The lag time between precipitation and reduction in salinity is approximately 10 h. Thus, apparently, both tide and precipitation influence the water in the basement of the SDP house.

#### 3.1.3. Jones House Time and Quantity Results

**Figure 9:**
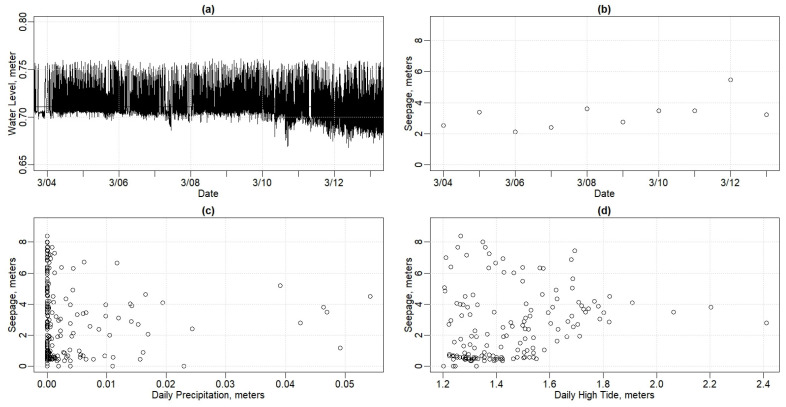
Graphs portraying Jones house. (**a**) Basement water levels from 3 March to 13 March 2024. (**b**) Daily average basement water levels vs. daily sum of precipitation. (**c**) Daily average basement water levels vs. daily high tides. (**d**) Daily average basement water level vs. daily high tides > 1.2 m.

The Jones house, approximately 240 m (800 feet) from the tidal Piscataqua River and set back 122 m (400 feet) from the SDP house, has an active sump pump. Therefore, “water level” in the basement is not sufficient to determine the level of flooding. On January 13, the sensor recorded the highest tide in our data record, where the water level exceeded the pump-on set level of approximately 0.77 m (2.5 feet) above MSL. Throughout the rest of the period of record, the sump pump reliably maintained basement water levels between 0.77 m and 0.64 m (2.53 feet and 2.09 feet) in elevation (Figure 9a). Thus, water levels do not correlate to groundwater intrusion. While we certainly can assert that the January 13 event was unusual, the data also imply that a metric other than simply maximum water level is needed to understand general flooding at the Jones house.

Daily water pumped (in meters and feet) was calculated for the Jones house basement by summing the depth by which water level was reduced per time step during a given day. The resulting value represents the hypothetical depth of water that would have arisen in the basement if the pump had not been present (Figure 9b). In the water level data, shown in Figure 9a, on the morning of 4 March, the lines are farther apart, indicating that less water is being pumped, and in the evening of 12 March, the lines are closer together, indicating that more water is being pumped. Translating that data to volume pumped Figure 9b demonstrates higher depth, and therefore higher volume pumped, on March 12. Note that the elevations recorded in the SDP and Jones houses are reported at the same datum (NAVD88). Thus, the water levels in the Jones house basement are consistently lower than those in the SDP house overall.

Figure 9c shows the depth of water pumped out of the Jones house basement as a function of the rainfall in the previous 24 h (R^2^ = 0.11). Dry periods, with rainfall less than 3.8 cm (1.5 inches) in the previous week, tend to have less than average water pumped. Wet periods, with rain above 5.1 cm (2 inches) in the previous week, tend to result in more water being pumped out of the Jones house basement. While the high tide impacts do not seem to help describe the scatter in the data, it is interesting that many of the wetter weeks, with 8 to 10 cm or 3 to 4 inches of rainfall for the past seven days, also had higher high tides than usual.

Figure 9d shows the depth of water pumped out of the Jones house basement as a function of tides higher than 1.2 m (4 feet). The Jones house basement water level does not show the strong linear relationship that the SDP house basement water level does (Figure 7d) for the tidal level driver.

**Figure 10:**
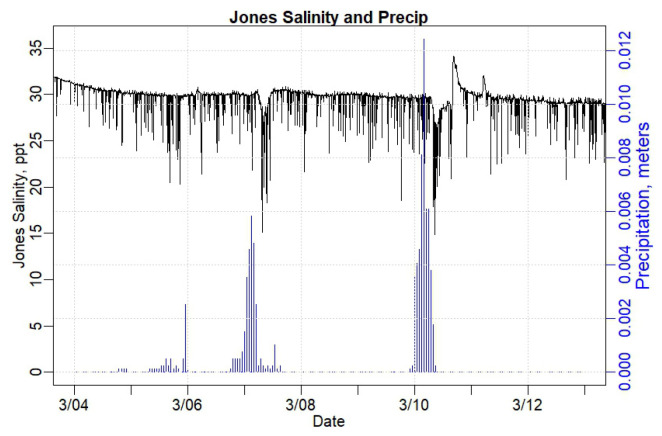
Graph portraying Jones house salinity levels (black, left axis) in basement water and precipitation (blue, right axis) measured at the Pease Air Force Base, Portsmouth, NH.

To further investigate the tidal influence on flooding in the Jones house basement, salinity levels were measured. Figure 10 shows the salinity in the Jones basement water for the same period as shown above in Figure 8 for the SDP house. The salinity in the Jones house tends to be 4 to 5 ppt lower than in the SDP house, lower than typical ocean water, and much more variable than the SDP house. The lag time between the precipitation and the reduction in salinity at the Jones house is approximately 5 h, much quicker than in the SDP house at approximately 10 h. Figure 10 also shows a downward trend in salinity throughout the period from 3 March to 14 March 2024, even though the high tides were generally increasing throughout this period (Figure 6).

Both rain and tides appear to impact the flooding in the Jones house, but in a less direct manner than seen in the SDP house. The lag time between higher high tide in the Piscataqua River and the peak of water seepage in the Jones house basement is approximately four hours, much greater than the approximate one-hour lag time to the SDP house.

### 3.2. Compound Flooding Model Results

#### 3.2.1. Shapley Drisco Pridham (SDP) House Model Results

The height of the tides in the nearby Piscataqua River influenced basement flooding in the SDP house in two ways. First, significant flooding does not occur until the height of the high tide is over 1.2 m (4 feet) above mean tide. Lower high tides of less than 1.2 m above mean tide, for instance, do not seem to trigger significant flooding in the basement. Second, when tides are above 1.2 m mean tide, there is a roughly linear increase in flooding in the SDP house basement. Table 1 shows results for both flooding vs. “all high tides” and flooding vs. “only high tides greater than 1.2 m”. Using the high tides only greater than 1.2 m results in a higher correlation coefficient (0.895 vs. 0.9406).

The Breusch–Pagan test describes the homoscedasticity vs. heteroscedasticity of the residuals. Homoscedasticity refers to the condition in which the variance of the residuals, or errors remaining once independent variables have been included, remains constant across all levels of the independent variables. This uniformity of errors suggests that the model’s predictive accuracy is consistent, regardless of the value of the predictors. In other words, the model is equally good at predicting the dependent variable throughout the range of the independent variables. In contrast, heteroscedasticity occurs when the variance of the residuals varies with the level of the independent variables, indicating that the model’s predictive accuracy may be less reliable for certain ranges of the independent variables. Identifying and addressing heteroscedasticity helps to ensure the robustness of statistical inferences drawn from the model.

Looking at only the days where the tides were greater than 1.2 m also results in a higher Breusch–Pagan *p*-value (1.29 × 10^−7^ vs. 0.3131). The relationship between flooding and high tides greater than 1.2 m is, therefore, homoscedastic. This means that the model’s residuals are consistent throughout the tidal range above 1.2 m. In other words, eliminating the lower high tides from the model also eliminated the range for which the model errors are not consistent.

The impact of rain on the flooding in the SDP house is more subtle than the impact of the tides, as Table 1 shows. Including rain only slightly increases the correlation coefficient of the model (from 0.895 to 0.8998 for all high tides, and from 0.9406 to 0.9408 for days with a high tide greater than 1.2 m). Including precipitation also does not seem to impact the homo/heteroscedasticity of the model at the Jones house. While including consideration of precipitation in the model can increase the R-squared value slightly, the change to the accuracy of the relationship is minimal. 

#### 3.2.2. Jones House Model Results

Table 2 summarizes the model results for the basement flooding in the Jones house. Both tides and precipitation are significantly related to flooding but with low R-squared values for all values. The additional complication of the active pump in the basement of the Jones house may influence the model results. Perhaps the groundwater is higher or more apt to enter the Jones house for some structural reason, making the rain and tides seem less important, but we cannot conclude this within the current study.

## 4. Discussion

### 4.1. Potential Benefits of Sensor Networks to Cultural Heritage and Preservation

Interest in monitoring and preserving cultural heritage is at the core of organizations such as UNESCO, the United States Department of Interior, state and private offices of culture and preservation, and public and private museums. The diversity, geographic distribution, and fragility of some cultural heritage sites can pose challenges for managers to monitor these locations regularly. However, the rise of smart sensors and wireless streaming sensor networks over the last decade can help to mitigate the challenges of monitoring these sites. Wireless streaming sensor networks, in particular, can be helpful to inform and allow managers to respond and preserve these locations in near-real time. Wireless sensor networks, once deployed, can reduce labor costs due to a decreased need for in-person inspections, with particular advantages for locations composed of multiple or geographically dispersed sites. Furthermore, wireless sensor networks, by reducing the number of needed in-person inspections, also have the potential to reduce damage to fragile sites by decreasing visits and treading. The addition of public-facing interfaces to such networks also have the potential for near-real-time data and easier dispersion of data for education and engagement purposes. 

### 4.2. The Network

Within this research, we fashioned and utilized a functioning end-to-end, wireless, streaming, environmental sensor network to better measure and understand compound drivers related to groundwater basement flooding at the Strawbery Banke Museum (SBM). To this end, our work supports Hypothesis H1 of this research, which states that “A wireless groundwater environmental sensor network can be built within the complex hydrological environment of the Strawbery Banke Museum coastal cultural heritage site”. However, we needed to take into account several important factors throughout the design and construction process of this network. These factors included the process of building a collaborative relationship with SBM and the City of Portsmouth to acquire permission to place sensor nodes on their properties. Permissions were granted through a series of meetings, demonstrations, and other communications with museum and city officials. Other factors that we needed to consider during the design and construction of the network included the costs of the equipment, choices of wireless transmission protocols, and power options for the network. The cost for equipment per sensor node ran between USD 3500 and USD 4500 each, and the kiosk equipment cost about USD 2000 in total. These and other labor and construction costs were balanced over two years as the project progressed and when the equipment was ready to be ordered and deployed. Wireless sensor data transmission protocol options available included cellular or Wi-Fi protocols because the Onset© data loggers used on the project could be purchased with either option. Although the SBM buildings do have a Wi-Fi network, we chose to transmit data to our cloud server via a cellular connection. We did this because the Wi-Fi connection also relied on a local AC-powered router that ran the risk of losing power immediately during power outages caused by storms or other events, but the cellular connection helped to mitigate this risk because the cellular equipment was powered by both an AC-power and an internal battery backup power source. This internal backup power source also helped to run the data loggers and sensors during power outages. This was an important consideration because storm events that may cause power outages also likely correspond in time with storm event related flooding when the sensor network can be of most useful to collect, transmit, and visualize data to keep museum and city officials aware of pending flood threats. This ability to maintain near-real-time data collection, transmission, and reporting of live flood data is a major advantage over older static, non-streaming sensor networks and is a major upgrade from the previous static sensor network that was deployed and utilized at SBM in 2012 [48]. Furthermore, the data collection cadence of 5 min in our current study is a considerable improvement over the 30 min data collection cadence utilized at SBM in 2012 [48]. This new cadence provides greater temporal precision to our current assessment results. 

### 4.3. Time and Quantities

Assessment of our collected network data showed distinct variations in timing and amounts of groundwater inundation levels and salinity levels at the Shapley Drisco Pridham (SDP) and Jones houses. Thus, our data supports Hypothesis H2 of our research that states, “Variations in the timing and quantities of groundwater intrusion levels and water salinity levels can be measured with a wireless latitudinal sensor network within the historic building basements at the Strawbery Banke Museum coastal cultural heritage site”.

The SDP house, located 122 m (400 feet) from the nearby Piscataqua River tidal estuary, showed no inundation during times of low tide and low high tides but saw higher peaks during higher high tide conditions, especially during times of storm surge and king tides, implying a strong tidal influence on the inundation. Peaks in inundation at the SDP house began approximately one-hour after the peak occurrences of the higher diurnal high tides over 1.2 m above mean sea level in the nearby tidal Piscataqua River. The Jones house, located 244 m (800 feet) from the nearby Piscataqua River tidal estuary, showed a pattern of low peaks and troughs of inundation within its basement as regular inundation was pumped out by the on-site sump pump over and over again. However, one spike in water inundation levels occurred at the Jones house during a concurrent king tide and rainfall event in mid-January of 2024, displaying a 4 h lag time from the peak of the king tide in the river, implying a tidal influence on inundation levels at the Jones house was strong enough to overwhelm the sump pump under extreme tide conditions. Other research studies have observed similar increases in groundwater levels with increases in tidal forcing [44,66] and decreases in tidal influence with increases in distance from the tidal source [67].

The SDP house, located closer to the river than the Jones house, exhibited regular salinity levels of 35 ppt, similar to typical saltwater salinities of 35 ppt [68]. The Jones house, located further from the river, was shown to have regularly lower salinity levels of 31 ppt (Figure 8 and Figure 10), indicating that the Jones house, though likely influenced by tidal forcing, may also be influenced more by fresh, terrestrial water sources than the SDP house. However, large drops in water salinity levels at both houses during storm events imply the presence of rainfall impacts in the flood waters for each. There was an approximately 10 h lag between peak rainfall and peak drops in salinity in the SDP house and an approximately 5 h lag between peak rainfall and peak drops in salinity in the Jones house, indicating that the Jones house may be more closely connected to the groundwater table or to surface water flooding. Other research studies have observed similar decreases in the salinity of groundwater with increases in distance to the tidal source [69]. However, as our sensor node in the Piscataqua River does not currently measure salinity, we cannot yet compare tidal river salinity directly with the salinity measured in the SBM houses.

Observed variations in the timing and amounts of groundwater inundation within our current study show similar patterns as those detected and reported as part of our 2012 static sensor study at SBM [48]. However, the additional collection and assessment of flood water salinity data in our current study helps to reinforce the influence that rain events play in the groundwater inundation levels and timing at both houses and implies the importance that compound flood drivers have within the complex hydrological environment at the SBM.

### 4.4. Compound Flood Models

The data that we have collected to date are already sufficient to show the strong influence of tides on SDP basement flooding and to make conjectures about the impact of tides on the Jones house, even though the salinity data are not completely understood. Water inundation model results for the SDP house show the strongest correlation (R^2^ = 0.9408) when using rainfall amounts and high tide levels greater than 1.2 m as input variables together. However, correlations varied from R^2^ = 0.1173 for rainfall alone and R^2^ = 0.9406 for high tide levels greater than 1.2 m when variables were modeled individually. These results support the idea that a simple model can help parse the levels of influence that tidal and rain event drivers have on compound groundwater basement flooding in the SDB house. The water inundation model results for the Jones house showed a stronger influence when using rainfall and tides greater than 1.2 m as input variables taken together (R^2^ = 0.172) than when rainfall (R^2^ = 0.2247) and tide levels greater than 1.2 m (R^2^ = 0.168) were used as variables individually. However, the low R-squared values of all model results provide us with less confidence in the model results. Therefore, our Hypothesis H3, which states, “Simple statistical models can be utilized to help parse levels of influence that tidal and rain event drivers have on compound groundwater basement flooding levels at the Strawbery Banke Museum”, is only partially supported. The inclusion of this kind of compound flooding model analysis in our current study is a complementary improvement to the analysis methods from our 2012 study [48]. Other research literature expounds on the benefits of compound flood models over univariate assessments [70]. Also, though larger-scale analyses, such as the Compound Flood Risk Assessments (CFRAs), can be useful for understanding flood risks at regional or watershed scales [71,72], we believe that analyzing the impacts on single buildings or campuses of interest may provide a more localized and focused assessment. However, the use of CFRA may be important for better prioritization of historic preservation sites across watersheds, cities, counties, states, or regions and could help play an important role in the designing of mutual aid programs between preservationists.

### 4.5. Public-Facing Interfaces

In order to complete our end-to-end wireless sensor network, we constructed a public-facing web interface to disseminate and visualize our collected data, engage the public, and support community decision making. This interface is similar to other project water-level flood data interfaces such as the Southeast Coastal Ocean Observing Regional Association (SECOORA), Southeast Water Level Network (SWLN) interface [40], and the USGS National Water interface [41], which all allow plotting of their data site locations on a web-map and dissemination and graphing of their current and historical data. However, the new SBM interface also provides a time-slider tool and animated water-level graphics within our web-mapping interface for stronger visualizations of how water levels change over time and space relative to each other. This interface is now used regularly by museum officials to review the quantity and frequency of groundwater inundation events within the SDP and Jones houses to help inform and improve their future preservation efforts. Museum officials also used this tool to monitor groundwater inundation in near-real time during the 15 January king tide/rainfall compound flooding event. Furthermore, different from other water level sensor interfaces, the SBM interface was ported for additional use as an outreach and education tool on a public touchscreen kiosk as part of the SBM’s Water Has a Memory exhibit. This is an important use of this technology with great potential for public outreach because SBM welcomes over 100,000 visitors to its campus annually. These efforts that support engagement and community decision making support Hypothesis H4 of our research, which states, “An interactive public facing interface can be built to support public engagement and local decision making with regards to groundwater inundation at the Strawbery Banke Museum”.

### 4.6. Recommendations for Strawbery Banke Museum

Now that the Strawbery Banke Museum (SBM) end-to-end wireless sensor network is up and running and our initial model assessment is complete, we have compiled the following list of recommendations for the current site assessment. These, in no particular order, include (1) monitoring meteorological forecasts of both tides and precipitation due to their potential as groundwater inundation drivers at the SBM, (2) monitoring groundwater intrusion in near-real time within the SDP and Jones houses with the use of the new SBM sensor network and its web interface while keeping in mind the potential that occasional power outages have on the collection and distribution of the data, (3) maintaining basement sump pumps regularly to keep them in working order for use during times of groundwater inundation, (4) mitigating impacts of future sea level rise and other compound flood drivers by ensuring that sensitive collections, artifacts, and materials are out of reach of flood waters where possible, (5) utilizing humidity sensors within groundwater affected historic buildings to better measure the potential effects of humidity on the buildings and their collections, and (6) employing additional sensors to better understand the depth and variation of the local water table relative to the Jones house to better understand the regular groundwater seepage within its basement.

### 4.7. Future Work

Future efforts to improve this research can include the expansion of the SBM environmental sensor network to a larger study area, an increased collection cadence of sensor measurements, the collection and assessment of a more varied set of environmental variables, improvements to the environmental model, and upgrades in functionality to the public-facing interfaces.

The expansion of our network can include placing sensors in other buildings on the SBM campus and across the south end of the City of Portsmouth to better understand the directionality and flow of surrounding groundwater. This expansion will allow for better engagement with local homeowners and others interested in preserving local historic properties. The expansion of the network can also include the placement of new salinity and water level sensors up and downstream on the Piscataqua River to better capture data about flood drivers. Increasing the collection cadence of sensor data to 1 min time steps may provide a better understanding of how groundwater inundation is occurring at a finer temporal precision. This may be particularly important at the Jones house, where there is a continuous seepage that keeps its sump pump regularly active. Additionally, the incorporation of new sensor types to our network also can help us to collect, report, and assess sump pump usage cycles and building humidity’s relative to groundwater inundation levels. Furthermore, the placement of a weather station on-site at Strawbery Banke can help us collect more localized measurements of rainfall, wind speed, and wind direction. These new sensor placements, cadences, and sensor types will play an important role in increasing the geographic distribution, temporal precision, and variable depth of our future model analysis. We are also interested in the potential of utilizing more advanced groundwater models and AI modeling techniques trained from our collected data and generated results in the future. Future enhancements to our public-facing interface can include the addition of data distributions and visualizations for the expanded set of sensor nodes, as well as the collection of usage statistics for the web and kiosk dashboards. The addition of an e-mail or text message inundation alert system to our network will also allow SBM managers to react better to groundwater flood events as they are happening.

## 5. Conclusions

With the ongoing and increasing threats of climate change-driven storms, storm surges, and rising sea levels, scientists, property managers, preservationists, and policymakers are looking for new ways to monitor, assess, and model surface and groundwater flood events to better protect our coastlines, infrastructure, and coastal cultural heritage sites. Within our research, we expanded the design of a previous static groundwater level environmental sensor network at the Strawbery Banke Museum (SBM) in Portsmouth, NH, to collect and now “stream” both groundwater and salinity levels in basements on-site for this purpose. With the use of the data, we also explored the use of assessment methods and simple compound flooding models to understand better the component drivers of coastal flooding. Furthermore, our use of a new public-facing interface allowed us to better disseminate the results of our work for more informed local decision making.

This study was designed as a case study to better understand how to adapt to and mitigate the effects of ongoing groundwater flooding on-site. The study has met these goals and provided the site managers at the museum with important findings to support their real-world adaptation and mitigation strategies. For instance, knowing adjacent river tidal levels and lag times that propagate to basement flooding allows site managers time to ensure that local house sump pumps are in working order before the flooding occurs, thus avoiding potentially more flood damage than what would occur if the pump was not working. Additionally, knowing the levels of salinity of basement flood waters provides site managers with important information about what building materials to incorporate in their future maintenance strategies.

Further, the findings of our work provide strong justification for (1) the expansion of the current network and a more complex data model for use across a larger area of the city and (2) the deployment of similar sensor networks on other historic campuses. Case study or prototype pilot projects are often used in this way before laying down larger expenditures of time, money, and effort for larger projects. It is our hope that the methods, assessments, and results laid out here can help provide others with new knowledge to build similar networks for the preservation of, and the community engagement for, other cultural heritage sites around the world.

## Figures and Tables

**Table 1:** Linear model results for Shapley Drisco Pridham (SDP) house, basement water level.

Schema	R^2^	*p*-Value	Slope	BP Value	BP *p*-Value	Results
All high tides	0.895	2.2 × 10^−16^	0.751	27.88	1.28 × 10^−7^	Heteroscedastic
High tides > 1.2 m	0.9406	2.2 × 10^−16^	0.884	1.0174	0.3131	Homoscedastic
Weekly rain along	0.1173	9.31 × 10^−16^	0.239	3.554	0.0594	Homoscedastic
Rain and all high tides	0.8998	2.2 × 10^−16^	Rain: 0.050 Tide: 0.735	23.563	7.65 × 10^−6^	Heteroscedastic
Rain and high tides > 1.2 m	0.9408	2.2 × 10^−16^	Rain: −0.009 Tide:0.890	1.3809	0.5013	Homoscedastic

**Table 2:** Linear model results for Jones house, basement water levels.

Schema	R^2^	*p*-Value	Slope	BP Value	BP *p*-Value	Results
All high tides	0.0863	5.62 × 10^−6^	0.0809	18.555	1.65 × 10^−5^	Heteroscedastic
High tides > 1.2 m	0.168	3.36 × 10^−7^	0.1811	25.386	4.69 × 10^−7^	Heteroscedastic
Weekly rain along	0.0247	0.01688	0.038	10.2	1.41 × 10^−3^	Heteroscedastic
Rain and all high tides	0.0918	1.71 × 10^−5^	Tide: 0.075 Rain: 0.019	22.81	1.11 × 10^−5^	Heteroscedastic
Rain and high tides > 1.2 m	0.172	1.66 × 10^−6^	Tide: 0.169 Rain: 0.019	26.393	1.86 × 10^−6^	Heteroscedastic

## Data Availability

Project data are available for graphing and visualization through the project’s in-person interactive kiosk within the Strawbery Banke Museum’s “Water Has a Memory” exhibit in Portsmouth, New Hampshire, and online via the project’s website at https://sbm-sensors.sr.unh.edu/ (accessed on 10 October 2024).
